# Supplementary material for: MetaRibo-Seq measures translation in microbiomes
Source: Nat Commun. 2020 Jun 29;11:3268. doi: 10.1038/s41467-020-17081-z (PMC7324362; doi:10.1038/s41467-020-17081-z)
Supplement: Supplementary file 10 — Supplementary Data 7 [file 41467_2020_17081_MOESM10_ESM.zip › File2/Confidence_High_Taxonomy/332716_out.krona.html]

Javascript must be enabled to view this page.

members
magnitude
magnitudeUnassigned
count
unassigned
taxon
rank

332716\_out

149

2
149
superkingdom

1239
phylum
149

186801
class
149

order
149
186802

1898207

SRS020869\_contig\_number\_12767
species
1

541000
family
148

7
genus

SRS013476\_contig\_number\_37759SRS024331\_contig\_number\_contig-100\_846.194298SRS048981\_contig\_number\_contig-100\_62.63SRS077502\_contig\_number\_contig-100\_2045.74135SRS078419\_contig\_number\_9797SRS1055049\_contig\_number\_contig-100\_880.88568SRS143372\_contig\_number\_6479
1263
148


SRS011405\_contig\_number\_15210SRS015579\_contig\_number\_26010SRS015663\_contig\_number\_27165SRS016629\_contig\_number\_352SRS018133\_contig\_number\_7468SRS018313\_contig\_number\_7238SRS018351\_contig\_number\_contig-100\_798.109649SRS018817\_contig\_number\_23517SRS042628\_contig\_number\_14839SRS045826\_contig\_number\_20101SRS047014\_contig\_number\_18078SRS054905\_contig\_number\_25449SRS077194\_contig\_number\_9368SRS077641\_contig\_number\_contig-100\_624.108716SRS078242\_contig\_number\_contig-100\_886.155675SRS097889\_contig\_number\_22922SRS1041090\_contig\_number\_3208SRS1041137\_contig\_number\_14659SRS104311\_contig\_number\_34546SRS104400\_contig\_number\_46354SRS144362\_contig\_number\_31554SRS144714\_contig\_number\_13401SRS148319\_contig\_number\_15759SRS148424\_contig\_number\_21226SRS149181\_contig\_number\_22768SRS893172\_contig\_number\_11746SRS893270\_contig\_number\_11008SRS893279\_contig\_number\_3090SRS893373\_contig\_number\_23402SRS893378\_contig\_number\_20033SRS971276\_contig\_number\_22331
1160721
31
species

2293177

SRS011084\_contig\_number\_23675SRS014313\_contig\_number\_5051SRS019030\_contig\_number\_6275SRS142980\_contig\_number\_5560SRS143780\_contig\_number\_12497
species
5


SRS013965\_contig\_number\_30107SRS015431\_contig\_number\_17047SRS018427\_contig\_number\_31444SRS019068\_contig\_number\_75280SRS019808\_contig\_number\_18343SRS1041164\_contig\_number\_contig-100\_501.45016SRS104912\_contig\_number\_contig-100\_532.120455SRS1055083\_contig\_number\_8616SRS140492\_contig\_number\_28651SRS893170\_contig\_number\_8792
2293149
10
species


SRS016954\_contig\_number\_19920SRS024625\_contig\_number\_contig-100\_310.120125SRS1041033\_contig\_number\_12174
1637508
3
species


SRS018541\_contig\_number\_11649SRS053335\_contig\_number\_3000SRS104084\_contig\_number\_10846SRS1041157\_contig\_number\_6857
2293233
4
species

species
28
165186

SRS011239\_contig\_number\_11398SRS011302\_contig\_number\_20660SRS014855\_contig\_number\_1116SRS015694\_contig\_number\_14098SRS015782\_contig\_number\_41692SRS015854\_contig\_number\_10796SRS016989\_contig\_number\_17083SRS021948\_contig\_number\_26719SRS023914\_contig\_number\_22142SRS024009\_contig\_number\_2220SRS024492\_contig\_number\_3079SRS024663\_contig\_number\_1909SRS046717\_contig\_number\_contig-100\_333.62511SRS047044\_contig\_number\_11272SRS049712\_contig\_number\_21103SRS051031\_contig\_number\_31316SRS064276\_contig\_number\_37278SRS076804\_contig\_number\_17709SRS098644\_contig\_number\_31750SRS1055056\_contig\_number\_9094SRS143523\_contig\_number\_26204SRS147377\_contig\_number\_11517SRS147425\_contig\_number\_5808SRS147919\_contig\_number\_6990SRS149325\_contig\_number\_15252SRS893341\_contig\_number\_10911SRS893358\_contig\_number\_13237SRS971275\_contig\_number\_8151

41978

SRS012273\_contig\_number\_37826SRS013638\_contig\_number\_18130SRS014235\_contig\_number\_contig-100\_812.251150SRS014979\_contig\_number\_31332SRS015578\_contig\_number\_5594SRS016018\_contig\_number\_10729SRS016495\_contig\_number\_3762SRS017103\_contig\_number\_20860SRS017821\_contig\_number\_28929SRS019685\_contig\_number\_22778SRS020394\_contig\_number\_10822SRS022071\_contig\_number\_24519SRS022524\_contig\_number\_13782SRS024075\_contig\_number\_15975SRS043768\_contig\_number\_17665SRS045739\_contig\_number\_11434SRS048870\_contig\_number\_12645SRS049446\_contig\_number\_5144SRS050925\_contig\_number\_25886SRS050941\_contig\_number\_contig-100\_351.130402SRS050998\_contig\_number\_16949SRS051882\_contig\_number\_21300SRS052027\_contig\_number\_18755SRS063040\_contig\_number\_44998SRS063985\_contig\_number\_18678SRS065504\_contig\_number\_27205SRS074670\_contig\_number\_7202SRS075716\_contig\_number\_contig-100\_83.43556SRS076976\_contig\_number\_5437SRS077294\_contig\_number\_19021SRS077335\_contig\_number\_5075SRS097920\_contig\_number\_25492SRS098061\_contig\_number\_7825SRS098073\_contig\_number\_12575SRS101433\_contig\_number\_15528SRS1041038\_contig\_number\_15389SRS1041139\_contig\_number\_contig-100\_932.33753SRS104636\_contig\_number\_28937SRS142599\_contig\_number\_27126SRS142712\_contig\_number\_contig-100\_1321.1321SRS142781\_contig\_number\_9051SRS142890\_contig\_number\_30678SRS143722\_contig\_number\_1845SRS144183\_contig\_number\_40750SRS147139\_contig\_number\_26989SRS147614\_contig\_number\_2189SRS147766\_contig\_number\_contig-100\_1707.143111SRS148159\_contig\_number\_44281SRS149075\_contig\_number\_19545SRS150029\_contig\_number\_17041SRS893292\_contig\_number\_8871SRS893295\_contig\_number\_12253SRS893300\_contig\_number\_11518
species
53

2
species

SRS013940\_contig\_number\_16212SRS014923\_contig\_number\_39645
2293194

3
species

SRS019693\_contig\_number\_6349SRS054352\_contig\_number\_21092SRS075878\_contig\_number\_15191
2293221

species
2
2293178

SRS049773\_contig\_number\_3178SRS893369\_contig\_number\_10675
